# Dp71 Point Mutations Induce Protein Aggregation, Loss of Nuclear Lamina Integrity and Impaired Braf35 and Ibraf Function in Neuronal Cells

**DOI:** 10.3390/ijms231911876

**Published:** 2022-10-06

**Authors:** Claudia Ivette Rugerio-Martínez, Daniel Ramos, Abel Segura-Olvera, Nadia Mireya Murillo-Melo, Yessica Sarai Tapia-Guerrero, Raúl Argüello-García, Norberto Leyva-García, Oscar Hernández-Hernández, Bulmaro Cisneros, Rocío Suárez-Sánchez

**Affiliations:** 1 Laboratorio de Medicina Genómica, Departamento de Genética, Instituto Nacional de Rehabilitación-Luis Guillermo Ibarra Ibarra, Ciudad de Mexico 14389, Mexico; 2 Departamento de Genética y Biología Molecular, Centro de Investigación y de Estudios Avanzados del Instituto Politécnico Nacional, Ciudad de Mexico 07360, Mexico

**Keywords:** dystrophin Dp71, Duchenne muscular dystrophy, neuronal differentiation, pathogenic mutations, BRAF35, iBRAF

## Abstract

Dystrophin Dp71 is the most abundant product of the Duchenne muscular dystrophy gene in the nervous system, and mutations impairing its function have been associated with the neurodevelopmental symptoms present in a third of DMD patients. Dp71 is required for the clustering of neurotransmitter receptors and the neuronal differentiation of cultured cells; nonetheless, its precise role in neuronal cells remains to be poorly understood. In this study, we analyzed the effect of two pathogenic *DMD* gene point mutations on the Dp71 function in neurons. We engineered C272Y and E299del mutations to express GFP-tagged Dp71 protein variants in N1E-115 and SH-SY5Y neuronal cells. Unexpectedly, the ectopic expression of Dp71 mutants resulted in protein aggregation, which may be mechanistically caused by the effect of the mutations on Dp71 structure, as predicted by protein modeling and molecular dynamics simulations. Interestingly, Dp71 mutant variants acquired a dominant negative function that, in turn, dramatically impaired the distribution of different Dp71 protein partners, including β-dystroglycan, nuclear lamins A/C and B1, the high-mobility group (HMG)-containing protein (BRAF35) and the BRAF35-family-member inhibitor of BRAF35 (iBRAF). Further analysis of Dp71 mutants provided evidence showing a role for Dp71 in modulating both heterochromatin marker H3K9me2 organization and the neuronal genes’ expression, via its interaction with iBRAF and BRAF5.

## 1. Introduction

Duchenne muscular dystrophy/Becker muscular dystrophy (DMD/BMD) is a neuromuscular X-linked disorder characterized by severe, progressive and irreversible muscle-wasting, with prevalence of 19.8 per 100,000 live male births [1]. DMD is caused by mutations in the *DMD* gene that lead to the absence or dysfunction of dystrophin, the major DMD product in muscle [2]. The *DMD* gene consists of 79 exons and seven alternative internal promoters, which direct the expression of different tissue-specific dystrophin isoforms [3,4,5,6]. Dystrophins form a mechanical link between the actin cytoskeleton and the extracellular matrix through the conformation of the dystrophin-associated protein complex (DAPC), which includes α- and β-dystroglycans (α-DG and β-DG, respectively); α and β dystrobrevins; α, β and δ sarcoglycans; and syntrophins [7,8]. Genetic defects in some DAPC components lead to muscle-fiber loss and ultimately result in different types of muscular dystrophy [9].

Dp71 is the shortest isoform of the *DMD* gene but the most abundant dystrophin in the adult brain [10,11,12,13,14]. Dp71 is transcribed from a promoter located in intron 62, and it is structured in different domains: a unique actin-binding domain and the cysteine-rich and C-terminal domains, which are common to all dystrophins [15]. Dp71 is involved in several biological processes including cellular architecture, cell adhesion, ion and water homeostasis, cell division, nuclear architecture, excitatory synapse organization and neuronal differentiation [16]. Interestingly, mutations affecting Dp71 and Dp140 isoforms are associated with the presence of neurodevelopmental symptoms in one-third of DMD patients, including low IQ scores, inattention and hyperactivity, autism and epilepsy [17,18,19,20,21,22], which implies that Dp71 is determinant for important functions in the nervous system. Particularly, Dp71 participates in synapse organization and function in CA1 hippocampus [23,24] and have a role in the clustering of the water channel aquaporin-4 (AQP4) and the inwardly rectifying potassium channel Kir4.1 in retinal glial cells [25,26], and the clustering and maturation of glutamatergic receptors in hippocampal neurons [23,24]. Recently, Dp71 and β-dystrobrevin, a binding partner of dystrophins, have been associated with the high-mobility-group (HMG) proteins BRAF35/HMG20b and iBRAF/HMG20a [27]. BRAF35 and iBRAF are ubiquitous non-histone DNA-binding proteins that, during neuronal differentiation, are involved in the regulation of genes containing repressor *element*-1 (*RE1*) sites through the corepressor complex LSD1-coREST [28,29,30,31].

Several types of mutations have been identified in DMD patients; although large deletions are the most frequent, duplications, insertions, small deletions and point mutations have also been described [20,32,33]. Although the majority of dystrophin mutations lead to prematurely truncated nonfunctional proteins, there are some cases wherein a single amino acid substitution or in-frame deletion provokes the DMD clinical phenotype. C3340Y and E3367del mutations, in full length Dp427 dystrophin, were previously reported in patients displaying the classical DMD phenotype, with residual levels of the dystrophin Dp427 in muscular biopsies. The C3340Y mutation (C272Y in Dp71) is localized in the ZZ domain within the cysteine-rich domain, while the E3367del mutation (E299del in Dp71) is localized in a highly conserved region adjacent to the ZZ domain [34,35,36].

In this study, we analyzed the effect of C272Y and E299del mutations on the Dp71 function in order to explore the potential deleterious effects of short mutations that do not generate a truncated dystrophin. We found that the C272Y and E299del mutations influence the Dp71 structure, induce the formation of Dp71 aggregates and alter the nucleus area and circularity. Moreover, both mutations affect the subcellular distribution of the Dp71 binding partner β-dystroglycan and components of the nuclear lamina. Remarkably, we revealed a role of Dp71 in neuronal differentiation, as point mutations caused the mislocalization of the repressive histone mark H3K9me2, BRAF35 and iBRAF and altered the expression of synapsin. These results shed important light on the participation of Dp71 in neuronal differentiation and emphasize the functional relevance of Dp71 integrity.

## 2. Results

### 2.1. Dp71-C272Y and Dp71-E299del Mutants Form Aggregates in Neuronal Cells

**Figure 1:**
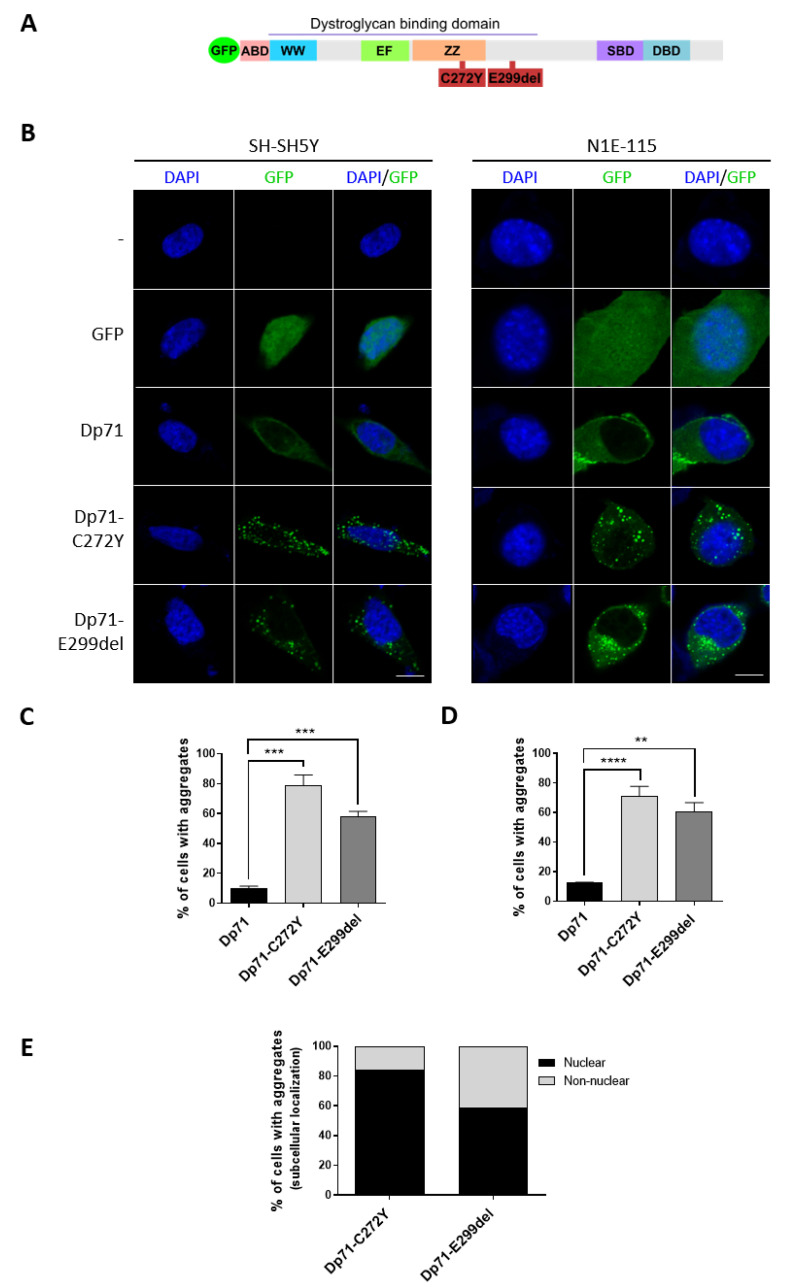
C272Y and E299del mutations provoke Dp71 aggregation in neuronal cells. (**A**) Schematic representation of Dp71 fused to GFP showing the location of C272Y and E299del mutations. The different Dp71 domains are depicted: WW, EF (EF hands), ZZ, SBD (syntrophin binding domain) and DBD (dystrobrevin binding domain). (**B**) SH-SY5Y and N1E-115 cells transiently expressing GFP, GFP-Dp71, Dp71-C272Y or Dp71-E299del were stained with DAPI to visualize nuclei, prior to being imaged by CLSM. Typical optical Z-sections are shown (scale bar = 10 μm). Percentage of (**C**) SH-SY5Y- or (**D**) N1E-115-transfected cells containing aggregates of Dp71 mutant variants is shown. (**E**) Quantification of the subcellular distribution of GFP-Dp71, Dp71-C272Y or Dp71-E299del aggregates in transfected N1E-115 cells. Nuclear, cells with aggregates observed in the nucleus; Non-nuclear, cells without aggregates observed in the nucleus. Results represent the mean +/- SEM of three independent experiments (n = 100). Significant differences were calculated using one-way ANOVA and Dunnett’s multiple comparison test (**, *p* < 0.01; ***, *p* < 0.001; ****, *p* < 0.0001 compared with Dp71).

To ascertain the effect of C272Y and E299del mutations on the Dp71 function, site-directed mutagenesis on the pEGFP-Dp71 vector was carried out (Figure 1A), and in-frame introduction of the mutations was verified by DNA sequencing (Figure S1). Then, the expression of GFP-tagged Dp71-C272Y and Dp71-E299del proteins was confirmed by Western blotting (Figure S1), and their subcellular localizations were further assessed by confocal laser scanning microscopy (CLSM) on N1E-115 and SH-SY5Y neuronal cells previously transfected with the corresponding vectors. Dp71 was found to be predominantly localized in the cytoplasm and to a much lesser extent in the nucleus (Figure 1B and Figure S2). Unexpectedly, aggregates of Dp71-C272Y and Dp71-E299del were found in most of the SH-SY5Y (78% and 57% respectively, Figure 1C) and N1E-115 (70% and 60% respectively, Figure 1D) neuronal cells, as well as in the epithelial HeLa cells (60% and 70% respectively; Figure S3A). Because the mutations are localized within (C272Y) or adjacent (E299del) to the ZZ domain, which mediates the nuclear import of Dp71 [37], we were prompted to analyze their potential effect on Dp71 nuclear targeting. Aggregates of both Dp71-C272Y (80%) and Dp71-E299del (60%) maintained the ability to enter the nucleus (Figure 1E). Interestingly, when endogenous Dp71 was immunostained for Dp71 in the presence of the Dp71 mutant variants, a clear decrease in nuclear immunolabeling intensity was observed compared with the nuclear signal observed in cells expressing wild-type Dp71 (Figure S3B,C). The above suggests that C272Y and E299del mutations have a dominant negative effect on endogenous Dp71 nuclear localization.

### 2.2. In Silico Predictions of Structure Changes in Dp71-C272Y and Dp71-E299del

**Figure 2:**
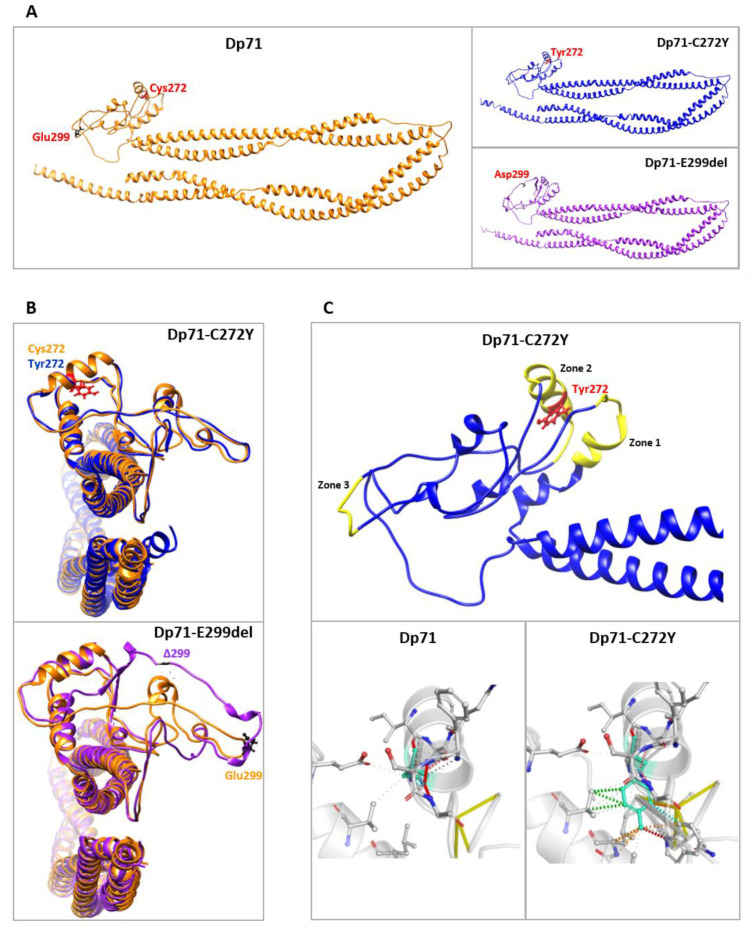
Structural analysis of C272Y and E299del mutations in Dp71. (**A**) Ribbon representation of the predicted Dp71 structures by I-TASSER server, illustrating the effect of C272Y and E299 mutations. (**B**) Superimposition of Dp71 (orange) with Dp71-C272Y (blue; upper panel) and Dp71 (orange) with Dp71-E299del (purple; bottom panel), highlighting the structural differences between Dp71 and its mutant variants. Protein models were visualized using Chimera v 1.16 (www.cgl.ucsf.edu/chimera/; accessed on 10 and 15 February 2022). (**C**) Upper panel: Ribbon representation of the effect of C272Y mutation on Dp71 structure. Amino acids are colored according to the vibrational entropy change upon mutation; yellow represents a rigidification of the structure in Dp72-C272Y mainly at positions 230 to 241 (zone 1), 265 to 280 (zone 2) and 295 to 300 (zone 3). Lower panels: Cys and Tyr amino acid residues at position 272 of wt and mutant Dp71, respectively, are colored in light green. In Dp71, hydrogen bonds are shown in red, while substitution by tyrosine in the C272 mutant causes a change in the surrounding interatomic interactions characterized by hydrophobic contacts (green) and weak hydrogen bonds (orange). Analyses were generated using the server DynaMut (www.biosig.unimelb.edu.au/dynamut/; accessed on 16 August 2022, The University of Melbourne and Instituto René Rachou Fiocruz Minas, sourced at Mexico City, Mexico) and Pymol Stereo 3D v. 2.5 4 (www.pymol.org, Schrödinger, sourced at Mexico City, Mexico), and the final models were visualized and edited using Chimera v. 1.16.1 (www.cgl.ucsf.edu/chimera/; accessed on 16 August 2022, University of California at San Francisco, sourced at Mexico City, Mexico).

To infer whether protein aggregates of C272Y and E299del mutants may be the result of structural changes on Dp71, protein modeling was carried out using the I-TASSER server (https://zhanggroup.org/I-TASSER/; accessed on 16 August 2022, The University of Michigan, sourced at Mexico City, Mexico). Predicted protein structures were generated in the absence of any crystal structure as a reference, and their stereochemical quality was assessed by Ramachandran plot analysis (https://zlab.umassmed.edu/bu/rama/; accessed on 16 August 2022, University of Massachussets Chan Medical School Zlab, sourced at Mexico City. Mexico). The accuracy of the predicted protein structures was 97–98% (Figure S4A,B). The selection of the best models was based on C-score and root mean square deviation (RMSD) values; the highest C-score in each series was −0.44 for Dp71, −0.45 for Dp71-C272Y and −0.43 for Dp71-E299del (Figure S4C). Ribbon representations of the predicted models for Dp71 and for C272Y and E299del mutants are shown in Figure 2A. We searched for structural differences between Dp71 and its mutant variants first using the TM-Align tool contained within the I-TASSER server (https://zhanggroup.org/TM-align/; accessed on 10 and 15 February 2022). Structural superposition of the predicted models showed that both cysteine 272 (Dp71) and tyrosine 272 (Dp71-C272Y mutant) are situated in a similarly arranged α-helix, while the lack of the E299 amino acid residue altered the structure of the loops in the vicinity of this deletion (Figure 2B). In line with this, a lower structural similarity score was obtained for Dp71-E299del (TM-score of 0.9523; RMSD: 1.74 Å) than for Dp71-C272Y (TM-score of 0.9824; RMSD: 1.24 Å), compared with Dp71. Furthermore, increased structural rigidity in three different protein regions was determined for Dp71-C272Y using the DynaMut server (http://biosig.unimelb.edu.au/dynamut/, www.biosig.unimelb.edu.au/dynamut/; accessed on 16 August 2022, The University of Melbourne and Instituto René Rachou Fiocruz Minas, sourced at Mexico City, Mexico), which may be due to the higher number of Tyr-based intercatenary bonds than those mediated by Cys (Figure 2C).

Additionally, molecular dynamics simulations (MDS) were performed with each model under energy minimization conditions to assess their structural stability based on the variation in RMSD values through a 100 ps period. Generally, the higher the RMSD value, the lower the structural stability. As can be seen in the MDS trajectories shown in Figure S5A, the wild-type Dp71 protein spent by 70 ps to reach a stabilized conformation, while the C272Y mutant was stabilized in a shorter time (40 ps) and thereafter; otherwise, the variant E299del seemed to be stabilized from 40–80 ps but displayed vibrational variability from 80 ps until 100 ps, suggesting a high structural instability. Despite the short time used in MDS, these data are in agreement with the lower structural similarity between E299del with Dp71 and the higher molecular rigidity of the C272Y mutant. In order to search for possible changes in the structural flexibility in the wild-type and mutant proteins, the root mean square float (RMSF) values were calculated in each residue by using 100 ps simulations at 300 k. These data are shown in Figure S5B and preliminarily indicate that, within the ZZ domain (positions 243–291), no significant changes occur, but at position 299 and neighboring residues in E299del mutant, a lower RMSF value (i.e., lower flexibility and higher stability) could be occurring. Likewise, a possibly stabilizing effect in E299del and particularly C272Y mutants at the α-1 syntrophin binding site (positions 363–413) could take place. On the other hand, the N-terminal (positions 1–7) and WW domains (positions 8–20) displayed higher flexibility upon C272Y and E299del mutations. Hydrogen bonds (H-bonds) are also important factors for protein stabilization; therefore, we looked at these interactions between the mutated positions of Dp71 (272 and 299) and adjacent residues. As shown in Figure S5C, the change of cysteine for tyrosine at position 272 decreased the H-bonds throughout the 100 ps simulation, and, worthy of note, the glutamate deletion at position 299 did not render H-bonds (Figure S5D), a feature consistent with the lower stability of the E299del mutant protein, as determined in these preliminary MDS analyses.

Overall, the data obtained from protein modeling, structural alignments and preliminary MDS studies suggest that both C272Y and E299del mutations are able to disturb the Dp71 structure by modifying the residue interactions at the surroundings of the mutation, which may ultimately contribute to overall changes in protein stability and possibly a trend for the formation of Dp71 aggregates.

### 2.3. Dp71-C272Y and Dp71-E299del Alter Different Morphology Parameters of Neuronal Cells

**Figure 3:**
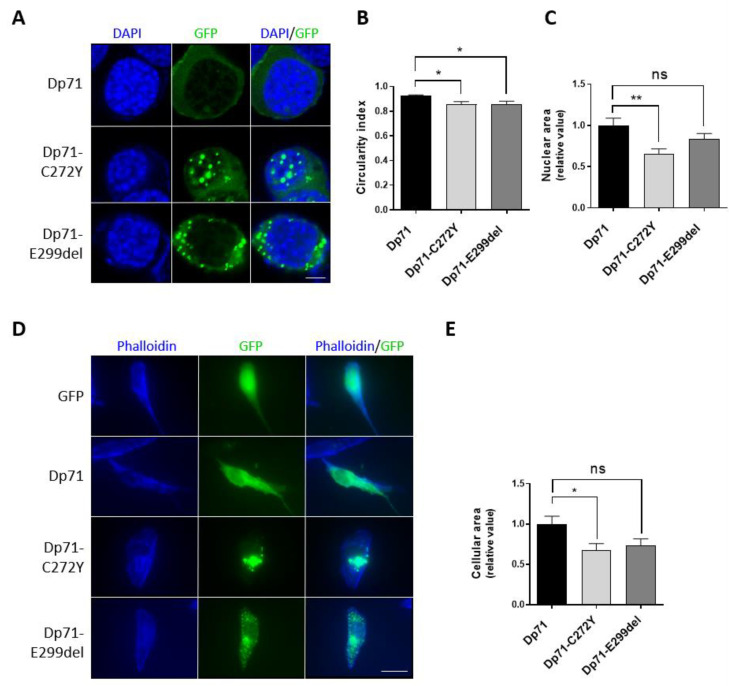
Effect of C272Y and E299del aggregates on neuronal cell morphology. (**A**) N1E-115 cells transiently expressing GFP, GFP-Dp71, Dp71-C272Y or Dp71-E299del were stained with DAPI to visualize nuclei, prior to being imaged by CLSM, and single typical optical Z-sections are shown (scale bar = 5 μm). Morphometric analysis of the nucleus was carried out to measure (**B**) nuclear circularity and (**C**) nuclear area using Fiji software. (**D**) SH-SY5Y cells transiently expressing GFP, GFP-Dp71 or Dp71-C272Y and Dp71-E299del were stained with phalloidin to visualize the actin-based cytoskeleton, prior to being imaged by epifluorescence microscopy (scale bar = 20 μm). (**E**) The cellular area was measured using ImageJ 1.53a software. Results represent the mean +/− SEM of three independent experiments (n = 100 cells per experimental condition), with significant differences calculated by one-way ANOVA and Dunnett’s multiple comparison test (ns, not significant; *, *p* < 0.05; **, *p* < 0.01).

Due to the structural role played by Dp71 on the nucleus and cytoskeleton [2,37,38], we reasoned that Dp71 mutant variants could alter the morphology of these subcellular compartments. To test this idea, the nuclear shape of N1E-115 cells expressing Dp71-C272Y or Dp71-E299del was assessed by staining nuclei with DAPI, while the cellular area of SH-SY5Y cells was measured by the phalloidin-staining of F-actin. Interestingly, a subtle but statistically significant decrease in nuclear circularity was found in N1E-115 cells expressing both Dp71 mutants, while a significant decrease in the nuclear area was only observed in cells expressing Dp71-C272Y when compared with those expressing GFP-Dp71 (Figure 3A–C). Similarly, the expression of Dp71-C272Y but not Dp71-E299del resulted in a clear decrease in the SH-SY5Y cellular area, compared with cells expressing GFP-Dp71 (Figure 3D,E). Overall, these results imply that the exogenous expression of Dp71 mutants is sufficient to disturb the morphology of neuronal cells.

### 2.4. The Distribution of Β-Dg Is Affected by the Exogenous Expression of Dp71-C272Y and Dp71-E299del

**Figure 4:**
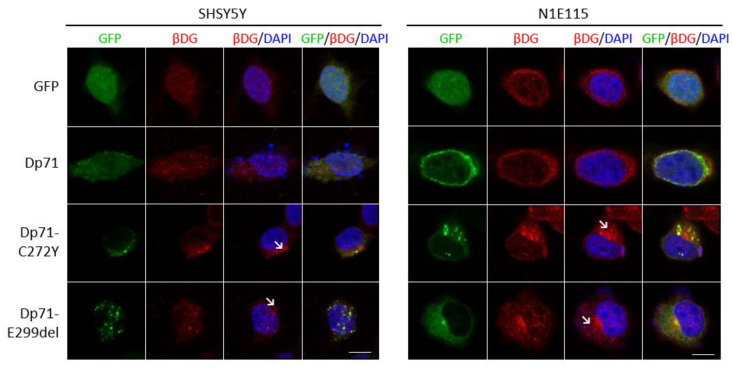
Neuronal cells expressing Dp71-C272Y and Dp71-E299del show the altered localization of β-DG. SH-SY5Y and N1E-115 cells transiently expressing GFP, GFP-Dp71, Dp71-C272Y or Dp71-E299del were immunostained for β-DG and counterstained with DAPI to visualize nuclei, prior to being imaged by CLSM. Representative optical Z-sections are shown (scale bar = 10μm). The polarized distribution of β-DG with aggregates is denoted by arrows.

Because the C272Y and E299del mutations are mapped on the β-dystrolgycan binding domain of Dp71 [39,40,41], we analyzed the impact of expressing these Dp71 mutants on the subcellular localization of β-DG. The distribution of β-DG was partitioned between the nucleus and the cytoplasm in SH-SY5Y/N1E-115 cells expressing GFP alone or GFP-Dp71; however, a polarized cytoplasmic distribution of β-DG with some aggregates was commonly observed in SH-SY5Y/N1E-115 cells expressing both Dp71 mutants (Figure 4). In line with previous reports [36,42,43], our results demonstrated that the dystroglycan binding domain integrity is relevant for the proper localization of β-DG.

### 2.5. Exogenous Dp71-C272Y and Dp71-E299del Expression Elicit the Mislocalization and Aggregation of Nuclear Lamins

Dp71 is involved in nuclear architecture maintenance through its association with lamins A/C and B1 [44]; therefore, we reasoned that the exogenous expression of Dp71-C272Y or Dp71-E299del could impair the distribution of nuclear lamins in neuronal cells.

**Figure 5:**
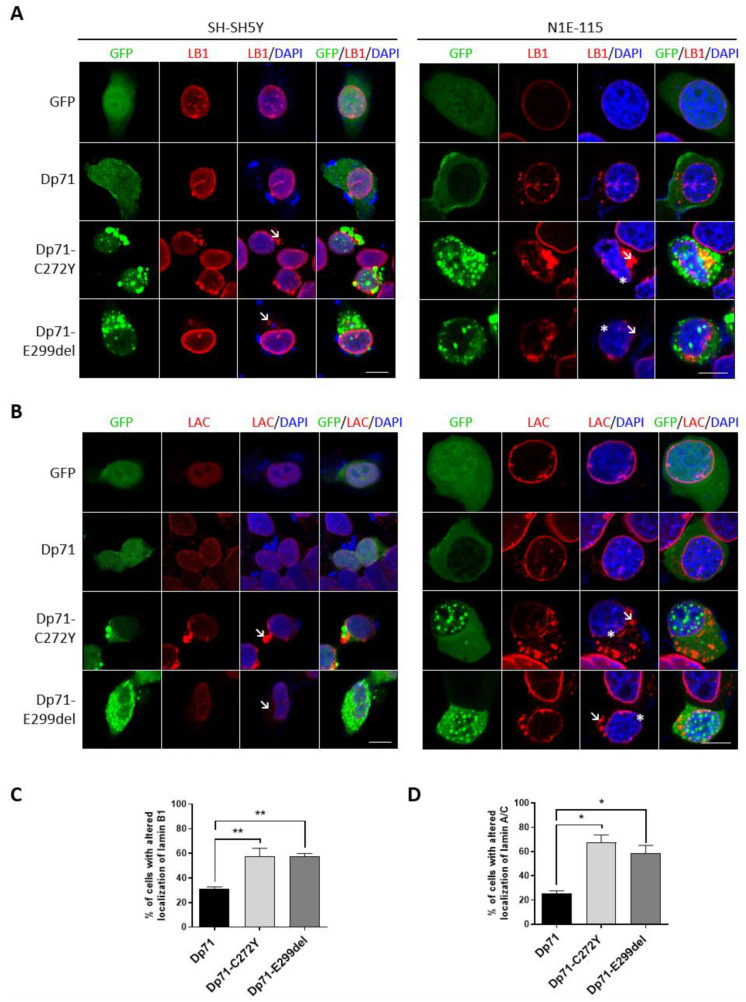
Neuronal cells expressing Dp71-C272Y or Dp71-E299del exhibit the altered localization of nuclear lamins. SH-SY5Y and N1E-115 cells transiently expressing GFP, GFP-Dp71, Dp71-C272Y or Dp71-E299del were immunostained for lamin B1 (LB1) (**A**) or lamin A/C (LAC) (**B**) and counterstained with DAPI to visualize nuclei, prior to being imaged by CLSM. Typical optical Z-sections are shown (scale bar = 10 μm). Cytoplasmic aggregates of nuclear lamins are denoted by arrows, and their faint discontinuous labeling is denoted by asterisks. The percentage of SH-SY5Y-transfected cells showing the aberrant distribution of lamin B1 (**C**) or lamin A/C (**D**) is shown. Results represent the mean +/− SEM of three independent experiments (n = 100 cell per experimental condition), with significant differences calculated using one-way ANOVA and Dunnett’s multiple comparison test (*, *p* < 0.05; **, *p* < 0.01).

In contrast with the typical ring-like nuclear staining of lamin B1 observed in N1E-115/SH-SY5Y cells expressing GFP or GFP-Dp71, an evident perturbation of its immunolabeling pattern was found in both neuronal cell lines when expressing Dp71 mutants. It was characterized by discontinuous labeling at the nuclear periphery and the presence of some patches and cytoplasmic aggregates (Figure 5A). Likewise, the exogenous expression of Dp71-C272Y or Dp71-E299del disrupted the localization of lamin A/C in N1E-115 and SH-SY5Y cells, resulting in a faint nuclear envelope labeling and the presence of prominent cytoplasmic aggregates (Figure 5B). Subsequent quantitative analysis showed that the percentage of SH-SY5Y transfected cells with aberrant lamin B1 or lamin A/C localization was significantly higher in cells expressing Dp71 mutant variants than in cells expressing GFP-Dp71 (Figure 5C,D). Collectively, these results imply that Dp71 mutant variants have a dominant negative effect on the nuclear lamina architecture, thereby disturbing their normal localization. 

### 2.6. Expression of Dp71-C272Y and Dp71-E299del Change the Distribution of the Heterochromatin Marker H3K9me2

**Figure 6:**
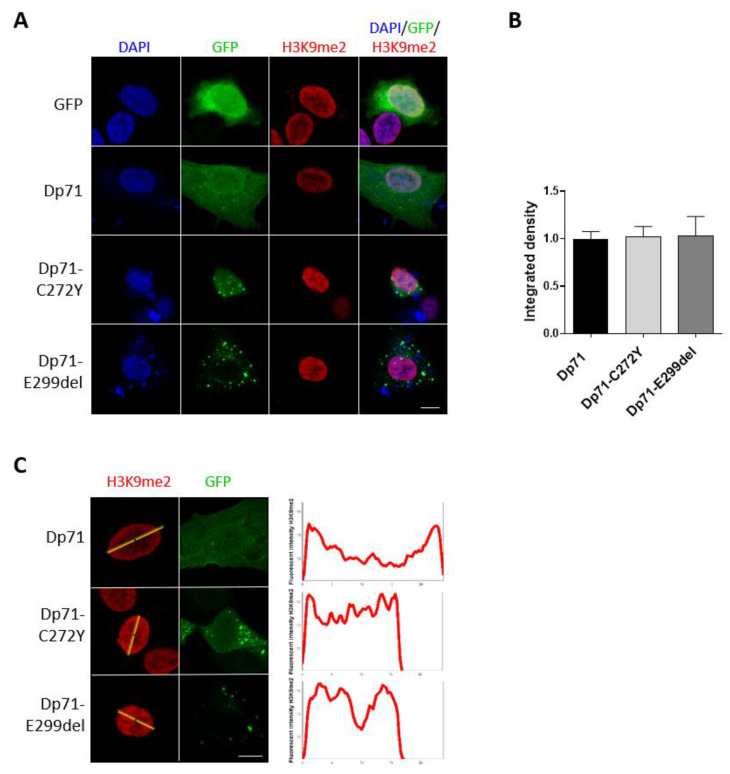
Distribution of the heterochromatin marker H3K9me2 is altered in SH-SY5Y cells expressing Dp71 mutant variants. (**A**) SH-SY5Y cells transiently expressing GFP, GFP-Dp71, Dp71-C272Y or Dp71-E299del were immunostained for H3K9me2 and counterstained with DAPI to visualize nuclei. Cells were imaged by CLSM, and representative single optical Z-sections are shown (scale bar = 10 μm). (**B**) Quantification of H3K9me2 immunofluorescence intensity was carried out in transfected cells using Fiji software. Results represent the mean +/− SEM of three independent experiments (n = 100 cells per experimental condition). Statistical analysis was performed using one-way ANOVA and Dunnett’s multiple comparison test. (**C**) Line profile analysis of H3K9me2 immunolabeling was carried out using digitalized images and Image J 1.53a software (n = 25 cells per experimental condition).

The connection of chromatin with the nuclear lamina takes place through the lamina-associated domains (LADs) [45,46]. LADs are commonly repressive environments enriched in the heterochromatin marks H3K9me2, H3K9me3 and H3K27me3 [46,47]. Thus, we ascertained whether the Dp71 mutant variants have the ability to interfere with the heterochromatin organization by analyzing H3K9me2 immunostaining in SH-SY5Y cells. The expression of Dp71-C272Y and Dp71-E299del shifted the immunostaining pattern of H3K9me2 from a prominent labeling at the nuclear periphery to a homogeneous distribution throughout the nucleoplasm, with no effect on its global immunolabelling intensity, as shown by CLSM and fluorescence intensity measurements (Figure 6A–C). Thus, the contribution of Dp71 to the functional organization of H3K9me2-enriched repressive chromatin domains, via its association with lamin B1, is impaired by the exogenous expression of Dp71 mutants.

### 2.7. Dp71 Is Involved in RE-1 Gene Expression through Its Association with BRAF35 and iBRAF

**Figure 7:**
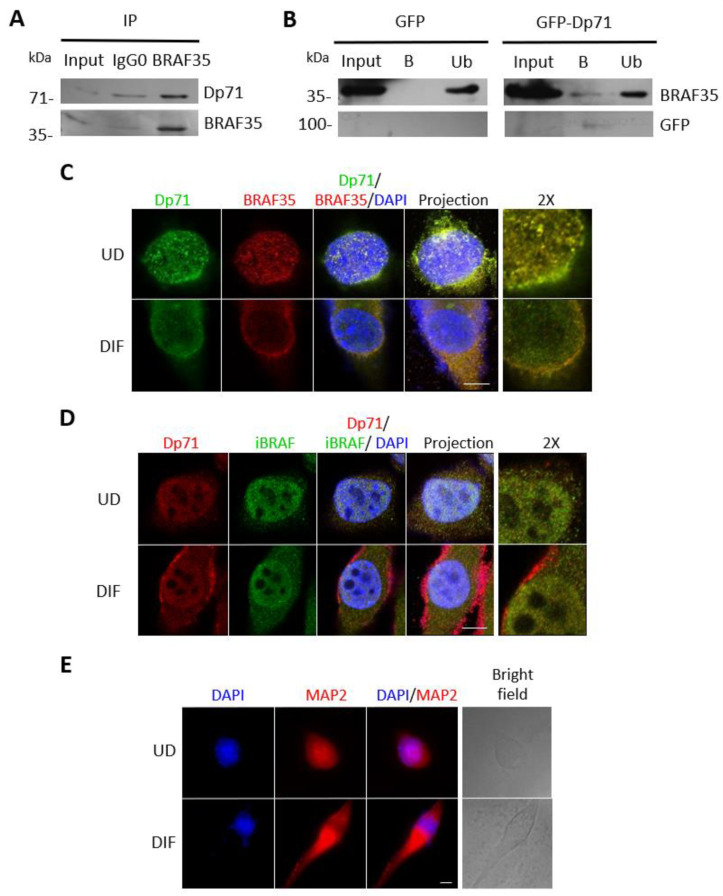
Dp71 interacts with BRAF35 and colocalizes with BRAF35 and iBRAF during the neuronal differentiation of N1E-115 cells. (**A**) N1E-115 cell lysates were subject to immunoprecipitation (IP) using anti-BRAF35 antibodies and the immunoprecipitated proteins were analyzed by Western blotting using antibodies against Dp71 and BRAF35. (**B**) Lysates from N1E-115 cells transiently expressing GFP or GFP-Dp71 were immunoprecipitated using the GFP-Trap system. The captured proteins were subjected to Western blot analysis using antibodies against BRAF35 and GFP. Input corresponds to 5% of protein lysates prior to immunoprecipitation; IgG0, nonspecific antibody; Ub, unbound proteins; B, bound proteins. Undifferentiated (UD) and differentiated (DIF) N1E-115 cells were immunostained with (**C**) anti-BRAF35 or (**D**) anti-iBRAF antibodies and anti-Dp71 and were antibody and counterstained with DAPI to visualize nuclei. (**E**) Undifferentiated and differentiated N1E-115 cells were immunostained for the neuronal differentiation marker MAP2 and counterstained with DAPI to visualize nuclei. Cells were imaged by CLSM, and representative single optical Z-sections are shown (scale bar = 10 μm).

Dp71 was found to interact in vitro with the high-mobility group (HMG)-containing protein 35 (BRAF35) and the BRAF-family-member inhibitor of BRAF35 (iBRAF) [27]. Both are components of a co-repressor complex that is required for the repressive action of the neuronal silencer RE-1 silencing transcription factor (REST), named LSD1-coREST [48,49]. Therefore, we were prompted to evaluate the functional relationship of BRAF35 and iBRAF with Dp71 in N1E-115 neurons. An immunoprecipitation assay using anti-BRAF35 antibodies revealed a specific interaction between endogenous BRAF35 and Dp71 (Figure 7A). In the inversed assay, GFP-Dp71, but not GFP alone, was able to immunoprecipitate endogenous BRAF35 (Figure 7B). Thus, Dp71 can bind to BRAF35 in N1E-115 neuronal cells. Moreover, abundant colocalization between Dp71 and BRAF35 was observed in the nucleus of undifferentiated N1E-115 cells (Figure 7C). Interesting, the coordinated redistribution of these proteins from the nucleus to the cytoplasm was observed upon DMSO-driven neuronal differentiation (Figure 7C). Likewise, a vast colocalization of Dp71 with iBRAF was observed in the nucleus of N1E-115 cells and to a much lesser extent at the cytoplasm. A subtle delocalization of both proteins from the nucleus to the cytoplasm occurred in response to differentiation, but the majority of the colocalization signal remained nuclear (Figure 7D). The differentiation of N1E-115 cells to the neuronal phenotype was confirmed by immunostaining for the neuronal marker microtubule-associated protein 2 (MAP2; Figure 7E) [50]. Overall, these data imply a spatial and functional relationship between BRAF35 and iBRAF with Dp71 during neuronal differentiation. 

**Figure 8:**
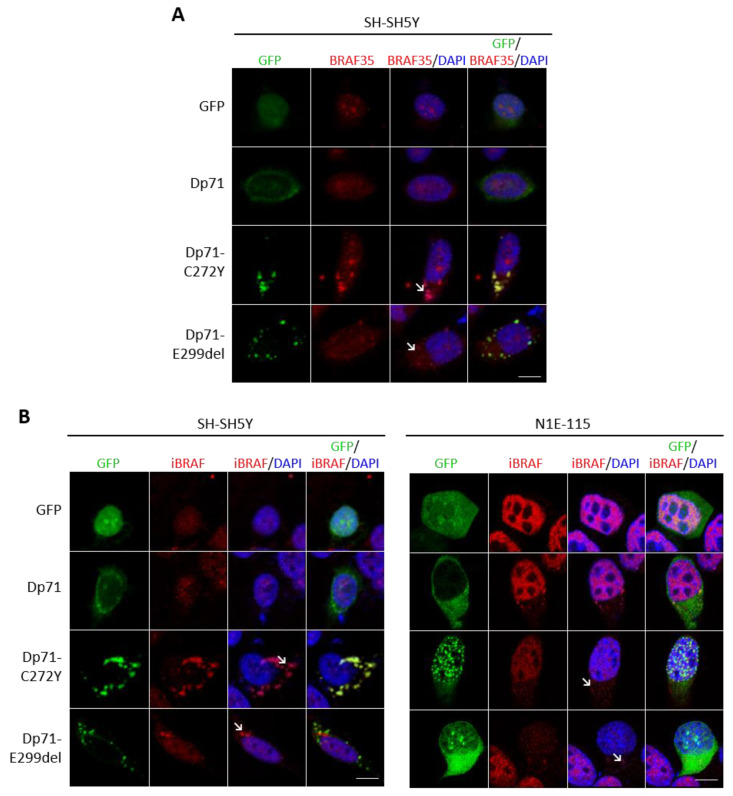
Cells expressing Dp71-C272Y or Dp71-E299del exhibit the altered localization of Braf35 and iBRAF. (**A**) SH-SY5Y cells transiently expressing GFP, GFP-Dp71, Dp71-C272Y or Dp71-E299del were immunostained for BRAF35 and counterstained with DAPI to visualize nuclei. (**B**) SH-SY5Y and N1E-115 cells transiently expressing GFP, GFP-Dp71, Dp71-C272Y or Dp71-E299del were immunostained for iBRAF and counterstained with DAPI to observe nuclei. Cells were imaged by CLSM, and representative single optical Z-sections are shown (scale bar = 10 μm). Cytoplasmic mislocalized BRAF35 or iBRAF is denoted by arrows.

To shed light on the biological significance of these interactions, we next investigated the effect of Dp71 mutants on the BRAF35 and iBRAF distribution in neuronal cells. Interestingly, the presence of aberrant cytoplasmic and nuclear aggregates of BRAF35 was evident in SH-SY5Y cells expressing either Dp71-C272Y or Dp71-E299del mutants (Figure 8A). Likewise, the exogenous expression of both Dp71 mutants induced the cytoplasmic aggregation of iBRAF in transiently transfected SH-SY5Y and N1E-115 cells (Figure 8B). It is worthwhile to note that a significant amount of BRAF35 and iBRAF aggregates were found to colocalize with those of Dp71 mutants. Overall, these results indicated that the association with Dp71 is required for BRAF35 and iBRAF to maintain proper subcellular localization and that Dp71 mutants impaired BRAF35 and iBRAF distribution, possibly by sequestering them into Dp71 mutant aggregates. 

**Figure 9:**
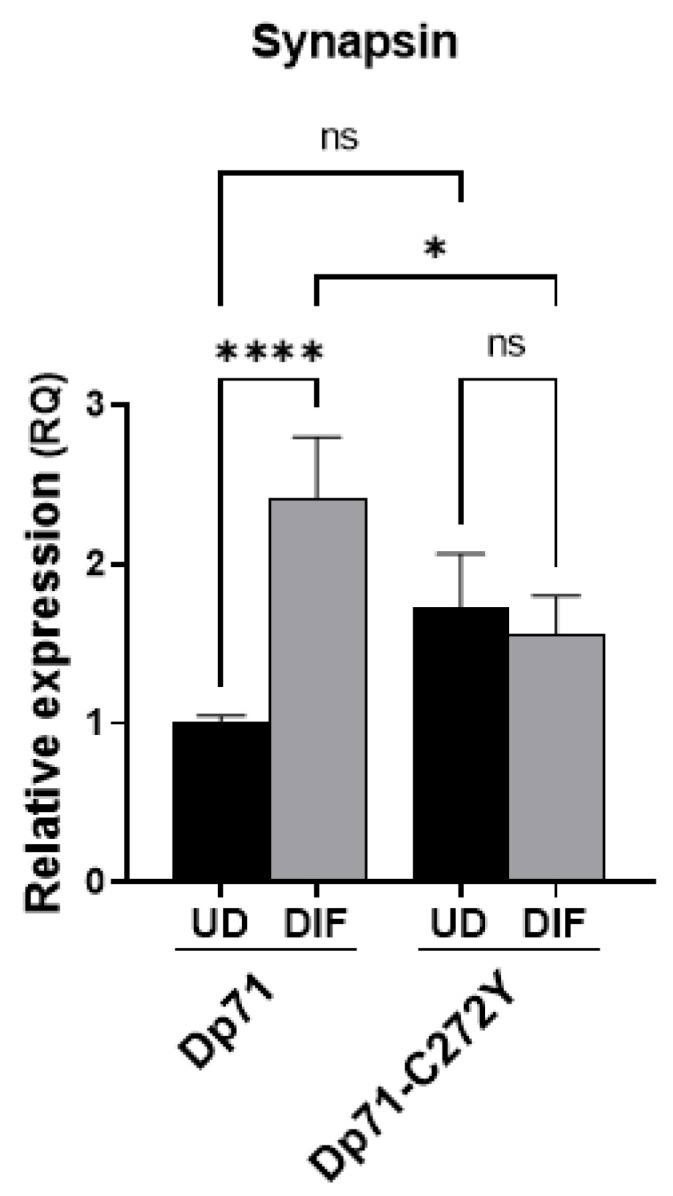
Dp71 but not Dp71-C272Y induces the expression of synapsin in neuronal cells. N1E-115 cells transiently expressing GFP-Dp71 or Dp71-C272Y were induced to neuronal differentiation by 3 days of DMSO treatment. The expression of synapsin mRNA was analyzed by quantitative reverse transcription polymerase chain reaction (qRT-PCR) in the transfected undifferentiated (UD) and differentiated (DIF) cells. *TBP* was used as endogenous control. Data correspond to the mean ± SEM of three independent experiments, with significant differences obtained by one-way ANOVA and Sidak’s multiple comparison test (ns, not significant; *, *p* < 0.05, ****, *p* < 0.0001).

We presumed that mislocalization of BRAF35 and iBRAF due to the expression of Dp71 mutants may affect their function as transcriptional regulators of neuronal genes. To test this hypothesis, we analyzed the expression of synapsin, a LSD1-coREST responsive gene, in N1E-115 cells expressing Dp71 or Dp71-C272Y. Remarkably, synapsin mRNA levels increased upon the differentiation of N1E-115 cells expressing Dp71; nonetheless, the induction of synapsin was suppressed when the differentiated N1E-115 cells expressed Dp71-C272Y instead of Dp71 (Figure 9). Taken together, these results support the idea that Dp71 has a role in the regulation of neuronal genes directed by the LSD1-coREST complex, via its association with BRAF35 and iBRAF.

## 3. Discussion

Although large deletions are the most frequent mutations in patients with DMD (60–70%), short mutations, including small deletions or insertions and punctual mutations, have been reported in 20% of these patients [20,32,33]. In general, double mutations are rare cases, and the reported cases are restricted to long non-contiguous deletions and duplications [51]. The functional effect of the mutations on the patient’s phenotype is mainly determined by the reading frame rule, with frameshifting and nonsense mutations leading to a DMD phenotype, and in-frame mutations leading to a BMD phenotype [52]. Nowadays, the application of high-throughput screening methodologies allows the routine detection of *DMD* gene point mutations [53]; however, their impact on dystrophin expression and function is largely unknown. In this context, this study aimed to analyze the effect of two pathogenic *DMD* gene point mutations (C272Y and E299del) on the Dp71 structure and function in neuronal cells. We chose these mutations because they both cause the classic DMD muscular phenotype [34,35,54,55], and specifically, the C272Y mutation elicited mental retardation and the absence of the electroretinogram b-wave in a carrier patient [35,36]. Furthermore, the mutations are located in or adjacent to the ZZ domain (C272Y or E299del, respectively), which is required for both the binding of Dp71 to β-DG [43] and driving the Dp71 nuclear import [56].

Dp71 is the shortest *DMD* gene isoform but the most abundant dystrophin in the nervous system [2,10,11,12,13,14]. Owing to the fact that the majority of patients with mutations affecting the coding sequence of Dp71 exhibit cognitive impairment, this protein has been implicated in DMD neuropathophysiology [17,18,19,20,21,22]. Unfortunately, due to the unavailability of DMD patients’ brain tissue, the reports about the effect of mutations affecting Dp71 on its own protein levels are restricted to muscle tissues [2], and, in only one study on iPSC-derived neurons from a DMD patient with a mutation in the intron 70, reduced levels of Dp71 were reported [57]. Nevertheless, a growing body of evidence support the function of Dp71 in the nervous system: Dp71 is required for the clustering of both the water channel aquaporin-4 (AQP4) and the inwardly rectifying potassium channel Kir4.1 in retinal glial cells [25,26]; the role of Dp71 as scaffold protein is extended to the clustering and maturation of glutamatergic receptors in hippocampal neurons [23,24]. Furthermore, studies in the Dp71-null mice unveiled the contribution of Dp71 to synapse organization and function and neuronal excitability in the CA1 hippocampus [23,24]. Finally, Dp71 expression is regulated during the cAMP-mediated differentiation of rat astrocytes [58] and nerve growth factor (NGF)–driven differentiation of PC12 cells [59,60].

Interestingly, the exogenous expression of Dp71 mutants originated protein aggregates in neuronal cells, which were found to localize in the nucleus and the cytoplasm. Intriguingly, both Dp71 mutants maintained their ability to reach the nucleus in spite of the fact that C272Y and E299del mutations localize in or close to the non-classical NLS motif of Dp71 [56]. Indeed, altered nuclear shuttling and impaired binding to importins of a C272Y-contained ZZ domain construct were found in C2C12 muscular cells [56]. Further studies are needed to determine whether Dp71-C272Y and Dp71-E299del are still being recognized by the importins system to enter the nucleus or if they used an alternative nuclear import pathway. Predicted structures of the mutant proteins evidenced potential changes in the structure of Dp71 that might explain the observed protein aggregation: E299 deletion abrogated an α-helix located near the mutation, while C272Y substitution increased the structural rigidity in regions surrounding the mutation. With awareness of the short time used for the MDS analysis, our results suggest that the structures of C272Y and particularly E299del proteins display a significantly lower overall stability than wild-type Dp71, as inferred from higher changes in conformational vibrational trajectories (RMSD), changes in Cα backbone (RMSF) at defined domains and diminished (C272Y) or absent (E299del) numbers of H-bonds at the surroundings of positions 272 and 299. It is conceivable that the destabilizing effect of these mutations on the Dp71 structure could enable the formation of homo- or hetero-complexes; however, in this regard, further analyses using longer times in MDS analyses (e.g., >50 ns) along with in vitro structural studies using Dp71 and mutant proteins in isolated and purified forms are required.

Although none of the previous studies on C272Y and E299del mutations have reported the formation of Dp71 aggregates, it is worthwhile to note that those studies were carried out in the context of full-length dystrophin (Dp427) or in the context of truncated protein constructs, expressed in different cell lines [34,35,43,56]. Specifically, the C272Y mutation affected the solubility of a construct including the CR domain and the C-terminus of full-length dystrophin (Dp427) in HEK293 cells [36]. On the other hand, several missense mutations located in the actin-binding domain resulted in the decreased protein stability and solubility of Dp427, thereby provoking protein aggregation and a loss of function [61]. The possible co-aggregation between Dp71 mutant variants and other dystrophin isoforms remains to be studied, but as they share the C-terminal, we hypothesize that this could be possible. Thus, we evaluated for the first time, to the best of our knowledge, the functional effect of these point mutations in the context of Dp71.

The dual subcellular localization of Dp71 confers on it the ability to interact with key cytoplasmic and nuclear protein partners and thereby modulate different cellular processes, including cell signaling and adhesion, neuronal differentiation and cell division [16,44,62,63,64,65]. Therefore, we analyzed whether Dp71 aggregates might disturb the distribution and function of Dp71 interactors. Consistent with this notion, we demonstrated that the exogenous expression of Dp71 bearing C272Y and E299del mutations impaired to a certain extent the distribution of β-DG in neuronal cells. The ZZ domain is involved in the binding of Dp71 to β-DG [36,42,66], and specifically, the physical binding between Dp71 and β-DG was shown to be impaired by C272Y mutation [43]. Remarkably, the distribution of lamin A/C and B1 was drastically perturbed in neuronal cells expressing either of the two Dp71 mutants: the typical ring-like nuclear labeling of lamins was shift to a discontinuous staining around the nucleus, and the presence of prominent patches and aggregates localized at the nuclear envelope and cytoplasm. Since Dp71 associates with lamins A/C and B1 to contribute to maintain the nuclear architecture [37,38,44,56,67], we hypothesized that the ectopic expression of Dp71 mutants acquires an aberrant dominant function that interferes with or displaces the endogenous Dp71, thereby affecting its interaction with the nuclear lamina of neuronal cells. One of the critical tasks of the nuclear lamina is the organization of peripheral chromatin through its interaction with LADs, which are enriched in histone repressive modifications, such as H3K9me2 [46]. In fact, alterations in nuclear lamina components lead to severely defective nuclei, with reduced H3K9me3 in the nuclear periphery [68,69]. H3K9me2 is associated with the epigenetic repression of neuronal genes mediated by the LSD1-coREST complex [48,49]. In line with an impact of Dp71 mutants on nuclear lamina function, a redistribution of H3K9me2 from the nuclear periphery to the nucleoplasm was noted in neuronal cells expressing Dp71-C272Y or Dp71-E299del. Therefore, Dp71 could contribute to the chromatin organization through its association with the nuclear lamina.

In this study we used two neuronal cell models, SH-SY5Y and N1E-115, which exhibited similar results but to a different extent, probably influenced by background differences in organism and tissue origin, as well as growth properties and adherence capacity. The last is important due to Dp71 having a role in cell adhesion via its association with β1-integrin [64]. Dp71 has been involved in neuronal differentiation [25,62,65,70,71,72]; however, its precise role on this process remained unclear. In this regard, the in vitro interaction of Dp71 with the LSD1-coREST complex components BRAF35 and iBRAF was previously described [27]. BRAF35 mediates the repression of REST-responsive genes in non-neuronal cells and neuronal progenitors, while iBRAF acts contrariwise activating REST-responsive genes during neuronal differentiation [29,30,31]. Herein, we showed the colocalization of Dp71 with both BRAF35 and iBRAF in neuronal cells and also demonstrated its association with BRAF35 in vivo. The functional significance of these results was evidenced by the fact that the ectopic expression of Dp71 mutants altered the distribution of BRAF35 and iBRAF and that the expression of synapsin, a REST-responsive gene, was abrogated in cells expressing Dp71-C272Y. Thus, Dp71 may contribute to the regulation of neuronal gene expression, via its association with BRAF35 and iBRAF. We propose that Dp71 acts as a scaffold for the anchorage of proteins involved in chromatin remodeling and neuronal gene expression. Clearly, further studies are needed to dissect the participation of Dp71 in the LSD-coREST complex function, as well as in chromatin remodeling.

## 4. Materials and Methods

### 4.1. Plasmid Constructs

Mutant variants of Dp71, C272Y and E299del were generated by site-directed mutagenesis using the pEGFPN1-Dp71a vector as template [32]. The oligonucleotides used were as follow: C272Y forward 5′-ACATCTGCCAAAGC**TAC**TTTTTTTCTGGTCG-3′ and reverse 5′-CGACCAGAAAAAAA**GTA**GCTTTGGCAGATGT-3′; E299del forward 5′-TCCGACTACATCAGGAGATGTTCGAGACTTTGCC-3′ and reverse 5′-GGCAAAGTCTCGAACATCTCCTGATGTAGTCGGA-3′. Briefly, 50 ng of plasmid pEGFPN1-Dp71a was added to a PCR reaction containing 0.5 μL of Herculase II Fusion DNA polymerase (Agilent Technologies, Santa Clara, CA, USA), 1X Herculase II reaction buffer, 0.2 mM dNTPs, 10 pmol of each oligonucleotide and 50 μL of water. Following temperature cycling, DpnI treatment was performed to cleave parental DNA and improve the efficiency of the mutant plasmid screening. The reaction was used to transform Top10 competent cells, and the transformation mixture was further plated on LB kanamycin plates. 

### 4.2. Cell Culture and Transfection

HeLa and N1E-115 cells were cultured in Dulbecco’s Modified Eagle’s Medium (DMEM), while SH-SY5Y cells were cultured in Eagle’s Minimum Essential Medium (EMEM)/F12 medium, supplied with 10% fetal bovine serum (FBS), 100 U/mL penicillin and 100 μg/mL streptomycin. Cell cultures were maintained at 37 °C, in a humidified 5% CO_2_ cell incubator. N1E-115 neuroblastoma cells were differentiated into the neuronal phenotype by using DMEM containing 2% FBS and 1.25% DMSO [73]. Where indicated, cells were transfected with lipofectamine 3000 (Invitrogen, Carlsbad, CL, USA) following the provider’s protocol and analyzed 24 h post-transfection.

### 4.3. Antibodies

Specific primary antibodies against the following proteins were used: GFP (sc-9996), lamin A/C (sc-376248), MAP2 (sc-74421) and BRAF35 (sc-390813) (Santa Cruz Biotechnology, Santa Cruz, CA., USA); dystrophin (ab7164 and ab15277), lamin B1 (ab16048), H3K9me2 (ab1220) and HMG20A (ab83284) (Abcam, Cambridge, UK); Dp71 (+78Dp71; Genemed Synthesis Inc. San Francisco, CA, USA).

### 4.4. Immunofluorescence and Confocal Laser Scanning Microscopy

Cells plated on coverslips were fixed in 4% paraformaldehyde (PFA), permeabilized in 0.2% triton X-100 and blocked in 0.5% gelatin and 1.5% FBS, following standard procedures. Primary antibodies were incubated overnight at 4 °C. Cells were then washed with phosphate-buffered saline (PBS) and incubated for 1 h at 4 °C with goat anti-mouse IgG (H+L) FITC (626511), goat anti-mouse IgG antibody (H+L) Cyanine3 (A10521) or goat anti-rabbit IgG antibody (H+L) Cyanine3 (A10520) (Thermo Fisher Scientific. Inc., Waltham, MA, USA); goat anti-rabbit IgG antibody (H+L) fluorescein (FI-1000), horse anti-mouse IgG antibody (H+L) DyLighy594 (DI-2594) and horse anti-rabbit IgG antibody (H+L) DyLight 594 (DI-1094) (Vector Laboratories Inc. Burlingame, CA, USA) as appropriate. When indicated, filamentous actin was stained with phalloidin (A22281; Thermo Fisher Scientific. Inc., Waltham, MA, USA). Cell preparations were mounted on microscope slides with VectaShield with DAPI (Vector Laboratories Inc. Burlingame, CA, USA) and visualized with a confocal laser scanning microscope (LSM, Eclipse Ti Series, Nikon Corporation Healthcare Business Unit, Japan; LSM 880, Carl Zeiss, Germany) using an oil-immersion 63X objective. Analysis of digitalized images was carried out using Fiji software to determine immunofluorescence intensity, nuclear area and circularity or Image J 1.53a to determine cellular area and line intensity scan analysis. 

### 4.5. Immunoprecipitation

Total extracts (250 μg) were pre-cleared with recombinant protein G-agarose beads (Invitrogen, Carlsbab, CA, USA); then, beads were removed by centrifugation at 1250 × *g* for 5 min, and pre-cleared extracts were incubated overnight at 4 °C with the appropriate immunoprecipitating or irrelevant IgG0 antibody. Subsequently, the protein G-agarose beads were added and incubated at 4 °C overnight. The immune complexes were collected by centrifugation at 1250 g for 5 min, washed three times with 500 μL of wash buffer [50 mM Tris–HCl pH 8.0, 150 mM NaCl, 0.5% (*v*/*v*) Triton X-100 and 1 mM PMSF] and eluted by boiling in 10 μL of 2X Laemmli sample buffer [100 mM Tris–HCl pH 6.8, 4% (*w*/*v*) SDS, 20% (*v*/*v*) glycerol, 5% (*v*/*v*) 2-mercaptoethanol, 0.01% (*w*/*v*) bromophenol blue]. GFP fusion proteins were immunoprecipitated using the GFPTrap^®^ bead system (Chromotek, Germany) in accordance with the manufacturer’s instructions.

### 4.6. Western Blotting

Cell protein extracts (80 μg) were separated by 10% SDS-polyacrylamide gels and transferred to PVDF membranes using a Transblot apparatus (Bio-Rad, Hercules, CA, USA). Membranes were blocked for 1 h in TBS-T (10 mM Tris-HCl pH 8.0, 150 mM NaCl, 0.05% Tween-20) containing 6% (*w*/*v*) low-fat dried milk and then incubated overnight at 4 °C with the corresponding primary antibody. After three washes in TBS-T (10 mM Tris-HCl pH 8.0,150 mM NaCl, 0.05% (*v*/*v*) Tween-20), membranes were incubated with the corresponding peroxidase-conjugate secondary antibody (Abcam, Cambridge, UK) and developed using the Western Lightning Plus-ECL system (PerkinElmer, Waltham, MA, USA).

### 4.7. Quantitative Reverse Transcription PCR (RT-qPCR)

Total RNA was extracted from transfected N1E-115 cells, both undifferentiated and differentiated, using TRIzol Reagent (Invitrogen, Carlsdab, CA, USA) according to the manufacturer’s instructions. RT-qPCR was carried out on the StepOnePlus Real-Time PCR System (Applied Biosystems, Hammonton, NJ, USA), using the Power up SYBR Green Master Mix (Thermofisher Scientific, Waltham, MA, USA) in accordance with the manufacturer’s instructions. The expression level for *SYN* mRNA was normalized to *TBP* mRNA values and quantified by the 2^ΔΔCT^ method. Results represent the mean +/− standard error of the mean (SEM) of three independent experiments. Significant differences were calculated using one-way ANOVA and Sidak’s multiple comparison test. For the analysis of synapsin mRNA expression, the following primers were used: forward, 5′-AGCTCAACAAATCCCAGTCT-3’ and reverse, 5′-TGAAAGCTGAGACCATCCG-3’. 

### 4.8. Modeling and MDS analyses of Dp71, Dp71-C272Y and Dp71-E299del Structures

A Iterative Threading ASSEmbly Refinement (I-TASSER) server (https://zhanggroup.org/I-TASSER/; accessed on 16 August 2022, The University of Michigan, sourced at Mexico City, Mexico) [74] was used to predict the structure of Dp71a and its mutant forms C272Y and E299del. The confidence of each model was quantitatively measured by the C-score. The protein models obtained were stereochemically evaluated by Ramachandran plot analysis (https://zlab.umassmed.edu/bu/rama/, accessed on 22 February 2022) [75]. For protein structure comparison between Dp71 and its mutant forms, the predicted models were analyzed by the TM-Align tool (https://zhanggroup.org/TM-align/; accessed on 16 August 2022, The University of Michigan, sourced at Mexico City, Mexico) [76]. Further details about the effect of C272Y mutation on Dp71 flexibility were obtained by using the DynaMut server (http://biosig.unimelb.edu.au/dynamut/; accessed on 16 August 2022, www.biosig.unimelb.edu.au/dynamut/; accessed on 16 August 2022, The University of Melbourne and Instituto René Rachou Fiocruz Minas, sourced at Mexico City, Mexico) [77]. The visualization and edition of protein structures were performed in Chimera v 1.16.1 (https://www.cgl.ucsf.edu/chimera/; accessed on 16 August 2022, The University of California at San Francisco, sourced at Mexico City, Mexico) [78] and PyMol Stereo 3D v 2.5.4 (https://pymol.org/2/; accessed on 16 August 2022, Schrödinger, sourced at Mexico City, Mexico) [79]. MDS studies were performed using the GROMACS package release 2020 (Royal Institute of Technology and Uppsala University, sourced at Mexico City, Mexico) [80] with a OPLS all-atom force field [81]. The protein was solvated in cubic water boxes with spc216 water molecules, and sodium ions were added to neutralize the system. The steepest descent method was applied to minimize the energy requirements of the simulation system [82]. Prior to simulation, two phases of equilibration were applied to the systems for 100 ps each. The first phase of equilibration was conducted under an NVT (constant number of particles, volume and temperature) ensemble to stabilize the temperature followed by the NPT (number of particles, pressure and temperature) ensemble to stabilize the pressure and density. The production of the three structures were performed at a temperature of 300 K for 100 ps. Analyses of protein structures, such as RMSD, RMSF, radius of gyration and hydrogen bonds, were performed using GROMACS simulation package [83]. For hydrogen bond calculation, a donor–acceptor cutoff distance of 3.5 Å and an acceptor–donor–hydrogen-bond angle cutoff of 30° were examined. Structures and trajectories were analyzed using Visual Molecular Dynamics (VMD) v 1.9 (University of Illinois at Urbana-Champaign, sourced at Mexico City, Mexico), and the preparation of figures was carried out using xmgrace and Chimera v 1.16.1 (https://www.cgl.ucsf.edu/chimera/; accessed on 16 August 2022, The Unversity of California at San Francisco, sourced at Mexico City, Mexico).

## 5. Conclusions

We showed that C272Y and E299del mutations caused Dp71 aggregation, likely by altering its structure. In turn, protein aggregates perturbed the distribution of key protein interactors of Dp71, including β-DG and nuclear lamins. By analyzing these mutants, we confirmed the contribution of Dp71 to maintain the nuclear envelope organization and provided new evidence showing the role of Dp71 in regulating the expression of neuronal genes, via its association with iBRAF and BRAF5.

## Data Availability

Not applicable.

## Supplementary Materials

The following supporting information can be downloaded at: https://www.mdpi.com/article/10.3390/ijms231911876/s1.
