# Supplementary material for: Dp71 Point Mutations Induce Protein Aggregation, Loss of Nuclear Lamina Integrity and Impaired Braf35 and Ibraf Function in Neuronal Cells
Source: Int J Mol Sci. 2022 Oct 6;23(19):11876. doi: 10.3390/ijms231911876 (PMC9570083; doi:10.3390/ijms231911876)
Supplement: Supplementary file 1 [file ijms-23-11876-s001.zip › 20221008-ijms-1895163-supplementary.pdf]

International Journal of
Molecular Sciences

MDPI

Supplementary Figures

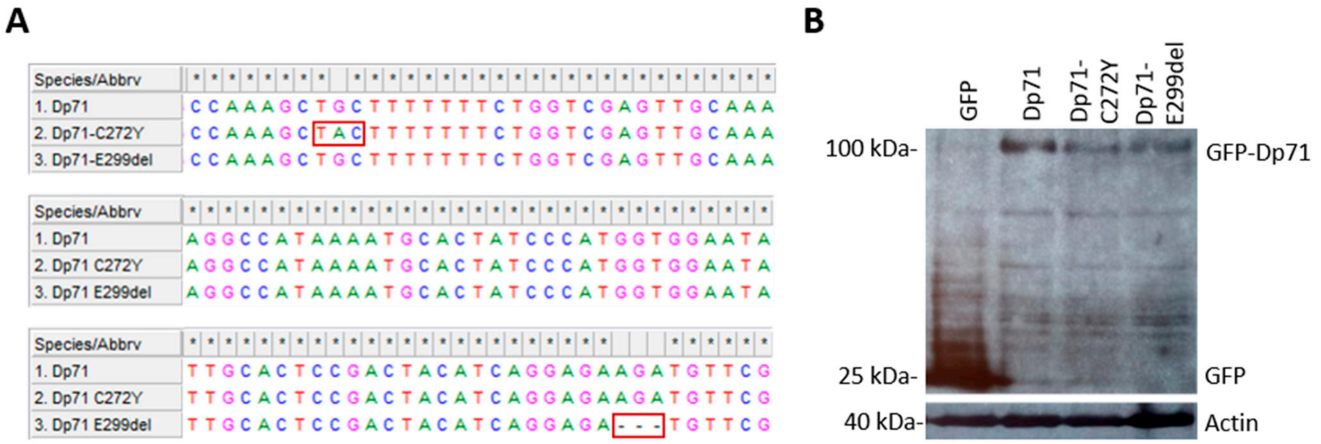

Figure S1. Characterization of C272Y and E299del mutant vectors. (A) Multiple sequence alignment showing the location of substitution for C272Y and elimination of E299. (B) Lysates from GFP, GFP-Dp71, GFP-Dp71-272Y or GFP-Dp71-E299del transfected N1E-115 cells were analyzed by western blotting using specific antibodies against GFP and actin (loading control).

International Journal of
Molecular Sciences

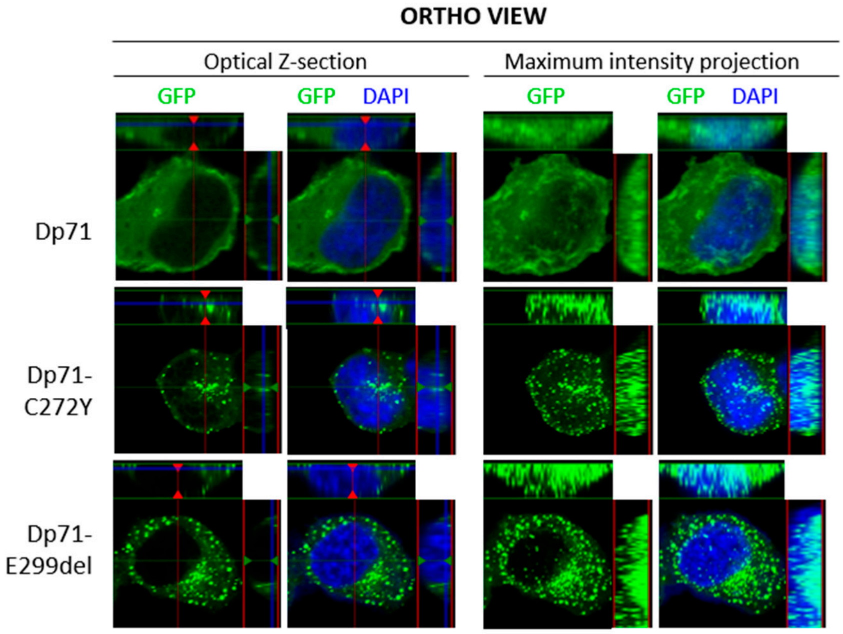

Figure S2. Subcellular distribution of GFP-Dp71, Dp71-C272Y and Dp71-E299del. N1E-115 cells transiently expressing GFP-Dp71, Dp71-C272Y or Dp71-E299del were stained with DAPI to visualize nuclei, prior to being imaged by CLSM. Ortho view of typical optical Z-sections and maximum intensity projections are shown. Ortho views were obtained by using the Zen 3.3 software by Carl Zeiss.

International Journal of
Molecular Sciences

MDPI

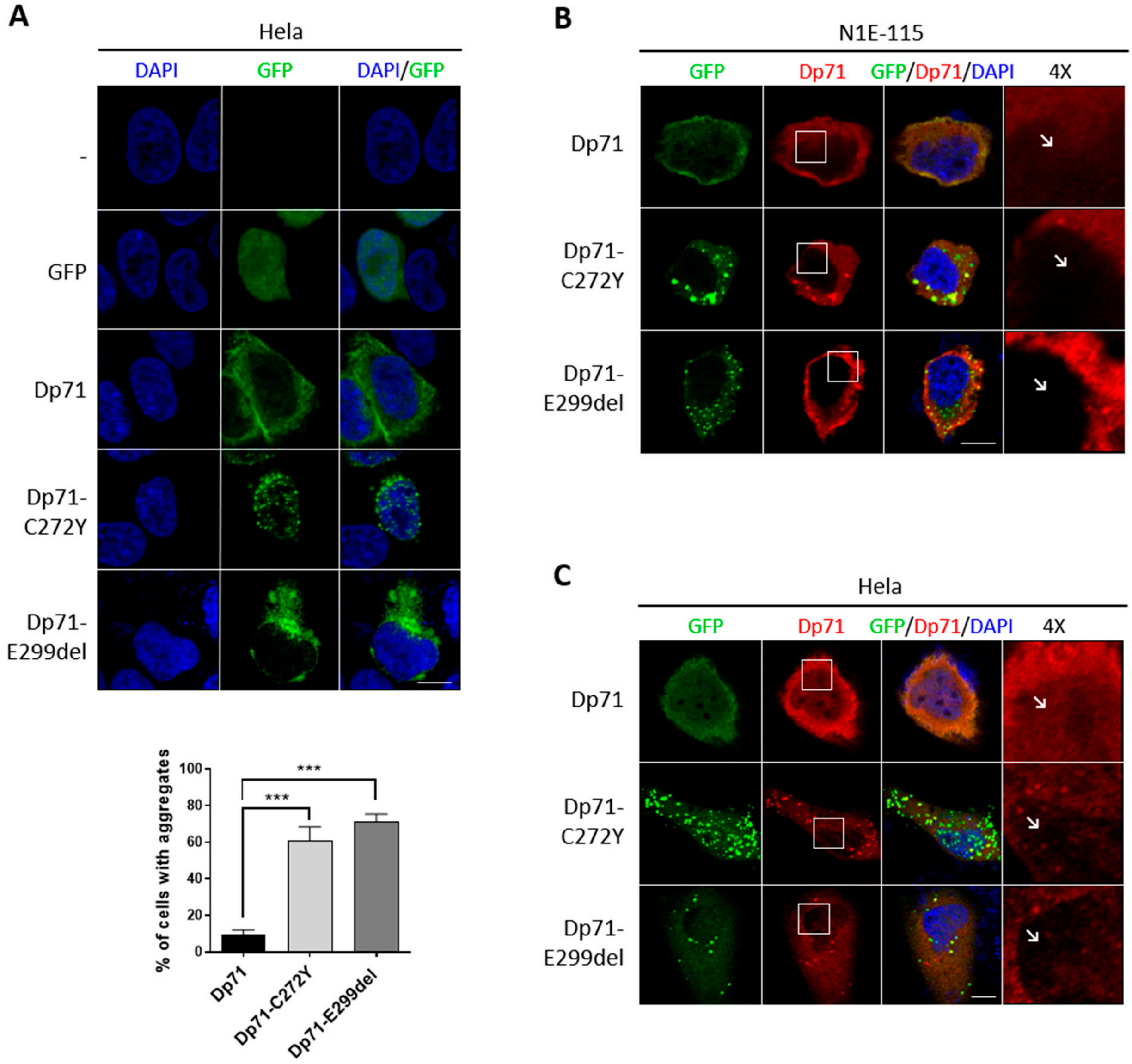

Figure S3. C272Y and E299del mutations cause the Dp71 aggregation in epithelial cell and alters the subcellular localization of endogenous Dp71. (A) Hela cells transiently expressing GFP, GFP-Dp71, Dp71-C272Y or Dp71-E299del were stained with DAPI to visualize nuclei. Cells were imaged by CLSM and representative single typical optical Z-sections are shown (scale bar = 10µm). Quantification of transfected cells that showed anormal distribution pattern of Dp71 (bottom panel). Results represent the mean +/- SEM of three independent experiments (n=100). Significant differences were calculated using one-way ANOVA and Dunnett's multiple comparison test (***, $p<0.001$ compared with Dp71). (B) N1E-115 or (C) Hela cells transiently expressing GFP-Dp71, Dp71-C272Y or Dp71-E299del were immunostained for Dp71 and counterstained with DAPI to visualize nuclei. Cells were imaged by CLSM and representative single typical optical Z-sections are shown (scale bar = 10µm). Note the absence in Dp71 signal in the nuclear area of mutant expressing cells (white arrows).

International Journal of *Molecular Sciences*

MDPI

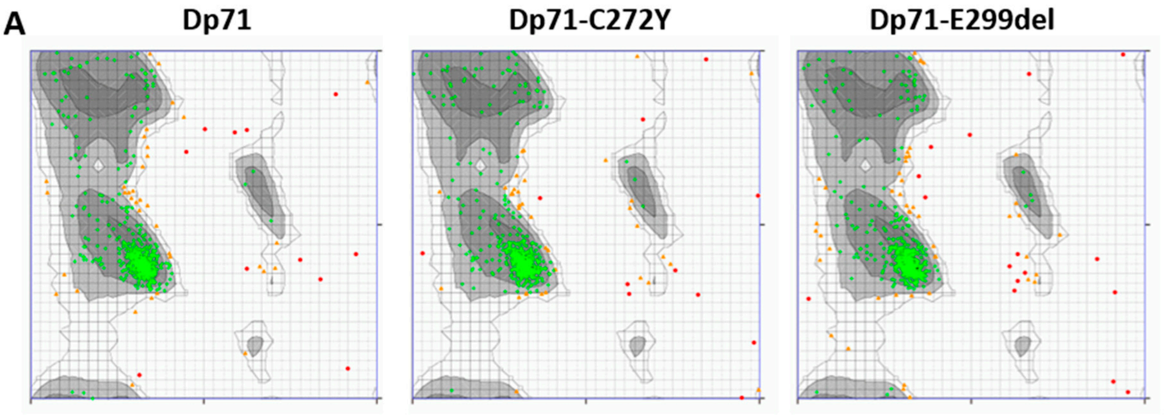

B

Model	Total	Residues in favored region (%)	Residues in allowed region (%)	Residues in outlier region (%)	Precision (%)
Dp71	602	92.19	5.98	1.82	98.18
Dp71-C272Y	602	92.19	5.98	1.82	98.18
Dp71-E299del	601	89.68	7.65	2.66	97.34

C

Model	C-score	RMSD (Å)	TM score
Dp71	-0.44	8.7 ± 4.6	0.66 ± 0.13
Dp71-C272Y	-0.45	8.8 ± 4.6	0.66 ± 0.13
Dp71-E299del	-0.43	11.4 ±	0.53 ± 0.15

Figure S4. Ramachandran analysis of modelled Dp71, Dp71-C272Y and Dp71-E299del. (A and B) Ramachandran analysis showed 92.19% (wtDp71), 92.19% (Dp71-C272Y) and 89.68% (Dp71-E299del) of residues present in favored region and 5.98% (wtDp71), 5.98% (Dp71-C272Y) and 7.65% (Dp71-E299del) in allowed regions, whilst only 1.82% (wtDp71), 1.82% (Dp71-C272Y) and 2.66% (Dp71-E299del) of residues were present in outlier region. All images were generated using Ramachandran Plot Server (https://zlab.umassmed.edu/bu/rama//). (C) Parameters of the models obtained in the I-TASSER server for the three models predicted.

International Journal of
Molecular Sciences
MDPI

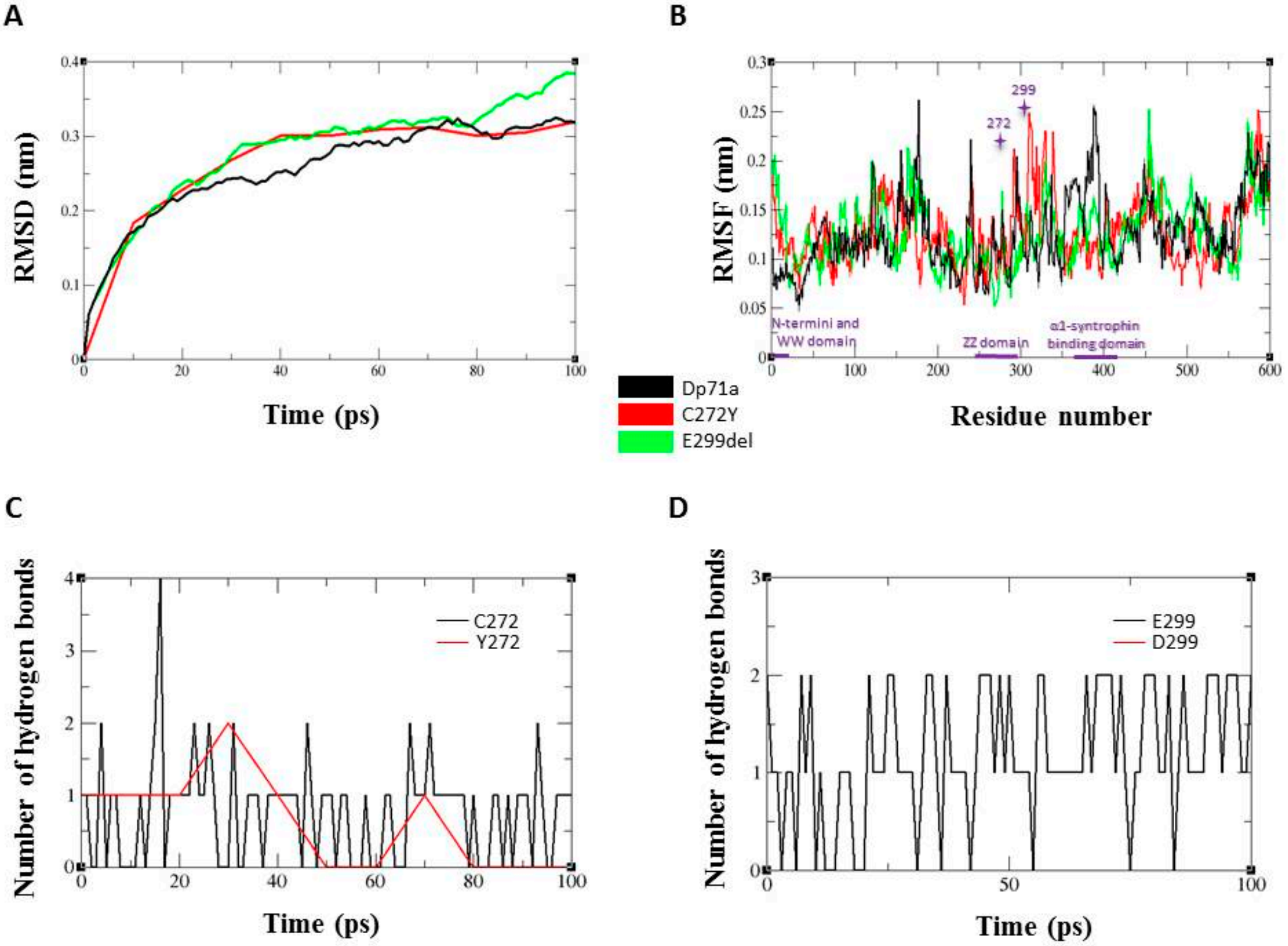

Figure S5. Molecular dynamics simulations of Dp71, Dp71-C272Y and Dp71-E299del protein structure models. (A) Trajectories of root mean square deviation (RMSD) values of the three proteins in a 100 ps simulation. (B) Root meand square float (RMSF) of C*α* backbone of residues of the three proteins through a 100 ps simulation. The positions with mutations are indicated in purple stars and domains of interest are denoted in purple bars. (C) Hydrogen bond interactions at the position 272 when occupied by cysteine or tyrosine during a 100 ps simulation. (D) Hydrogen bond interactions at the position 299 when occupied by glutamate or aspartate through a 100 ps simulation. In this case, none H-bound was found for aspartate.
